# Synthesize multiple V/H directional beams for high altitude platform station based on deep-learning algorithm

**DOI:** 10.1038/s41598-025-93251-7

**Published:** 2025-03-29

**Authors:** Korany R. Mahmoud, Ahmed M. Montaser

**Affiliations:** 1 Electronics and Communications Department, Faculty of Engineering, Helwan University, Cairo, 11795 Egypt; 2 National Telecommunications Regulatory Authority (NTRA), Giza, 12577 Egypt; 3 Electrical Engineering Department, Faculty of Technology and Education, Sohag University, Sohag, 82524 Egypt

**Keywords:** Deep learning, Deep neural network, Optimization algorithm, Antenna array, Beamforming technique, Engineering, Electrical and electronic engineering, Design, synthesis and processing

## Abstract

This paper investigates the integration of High-Altitude Platform Stations (HAPS) with Deep Learning (DL) models to enhance coverage capabilities. Recognizing the inherent limitations of traditional HAPS coverage, which is typically confined to a circular area, this work proposes a novel approach utilizing a 60-element Concentric Circular Array (CCA) operating at 2.1 GHz. To dynamically generate multiple vertical/horizontal (V/H) directional beams, the system integrates a Deep Neural Network (DNN) with a modified version of the Gravitational Search Algorithm and Particle Swarm Optimization (MGSA-PSO) algorithm. This hybrid approach optimizes the feeding phases of the CCA elements, enabling the system to effectively cover diverse road paths. Furthermore, the study incorporates realistic scenarios by utilizing the Computer Simulation Technology-Microwave Studio Suite (CST) with the Earth Explorer (EE) user interface tool to model real-world road paths, including those traversing challenging terrains such as rugged deserts with mountain chains and forested areas.

## Introduction

In response to the international goal of reaching a 0% broadband coverage gap, most governments in developing countries rushed into introducing technological developments and regulatory reforms to their telecommunications sectors, aiming to increase service penetration and connectivity. Meanwhile, with continued network expansion, the world’s coverage gap marginally closed to 350 million people (4% of the global population) in 2024, according to the Global System for Mobile Communications Association (GSMA) and International Telecommunication Union (ITU) reports^1,2^. Therefore, several technological solutions have emerged as the most cost-efficient alternatives to covering rural areas with broadband^3,4^. Satellite and aerial technologies are now at the top of the list of future solutions, promising to offer broadband connectivity to the hardest-to-reach places at a reasonable cost^5,6^. A comprehensive evaluation of broadband technology options for rural environments necessitates consideration of several key factors, as outlined in^7^. These factors encompass cost, technological capacity, target data rates, population density, socio-economic indicators (poverty, illiteracy, digital literacy), potential profitability, and the feasibility of suitable business models.

Research and businesses are increasingly interested in high-altitude platforms (HAPS) that operate in the stratosphere as a cost-effective broadband solution^8,9^. HAPS has the potential to significantly impact the telecommunications sector due to several key advantages, including (a) complementing terrestrial network operations by covering a larger surface area, (b) experiencing less interference, and (c) enabling faster deployment. According to the World Radio Communication Conference 1997 (WRC-97)^10^, HAPS are defined as telecommunications stations operating at altitudes between 20 and 50 km, maintaining a fixed point relative to the Earth. These platforms can operate autonomously while being remotely controlled from the ground and can be either manned or unmanned, encompassing airplanes, airships (essentially balloons), or other suitable vehicles^11–15^. HAPS can function for extended periods, often several months, without interruption, and provide connectivity over a substantial area (e.g., with a circumference of 200 km) utilizing solar power^16^. This approach offers the potential to create a cost-effective and environmentally friendly alternative to constructing expensive and underutilized terrestrial base stations, delivering comparable levels of coverage and capacity from the air. As a result, HAPS can support a diverse range of use cases in both developed and developing markets, including white spot reduction, emergency communications, disaster recovery, greenfield coverage, temporary coverage for events and tourist hotspots, private networks, connectivity for urban air mobility drones, and military communications^17^.

The allocations to the fixed service in the frequency bands 31–31.3 GHz and 38–39.5 GHz for global use by HAPS were selected by the WRC-19^18^. It further stated that administrations intending to deploy HAPS can use the existing global identifications for HAPS in the bands 47.2–47.5 GHz and 47.9–48.2 GHz anywhere in the world. The conference has also resolved that HAPS would utilize the frequency ranges of 21.4 to 22 GHz and 24.2 to 27.5 GHz for fixed service in Region 2. WRC-23 agenda item 1.4 is currently looking at taking into consideration, following Resolution 247 (WRC-19), the use of HIBS (High Altitude IMT Base Stations) within the mobile service allocations in particular frequency bands below 2.7 GHz, already identified for International Mobile Telecommunications (IMT), on a global or regional level, i.e., 694–960 MHz; 1710–1885 MHz; and 2500–2690 MHz. According to Resolution 221’s guidelines (Rev.WRC-07), the frequency bands 1885–1980 MHz, 2010–2025 MHz, and 2110–2170 MHz in Regions 1 and 3, and 1885–1980 MHz and 2110–2160 MHz in Region 2 are included in No. 5.388 A for HIBS use. In^17,19^ HAPS is suggested as a promising technology for rural coverage due to its extensive coverage capabilities. A single HAPS can provide 3G services over a large area, enabling cost-effective connectivity across multiple territories. One of the major advantages of the HAPS system is its capability to provide a higher elevation angle than terrestrial systems. Huge solar panels and energy storage systems can also be added to HAPS. Due to improvements in energy storage and solar panel efficiency, HAPS systems can operate for a long time with the required energy usage. Additionally, it’s claimed that the relatively fixed position of HAPS gives it an advantage over satellite technologies because satellites in Low Earth Orbit (LEO) travel at high speeds and can cover continents in a matter of minutes. As a result, while passing across oceans and less inhabited areas, some of the LEO satellites’ communication capacity is lost. On the other hand, HAPS systems’ generally stationary location avoids capacity wastage^20^. To bring 4th Generation Long-Term Evolution (4G LTE) and 5th Generation (5G) services to the sparsely inhabited areas of global wireless connectivity, there are numerous ongoing HAPS initiatives and inventions, including Google Loon, HAPS Mobile, and Airbus Zephyr^21–24^. In Arizona, United States, Airbus Defense and Space has successfully finished a new test flight campaign for its Zephyr HAPS. Two Unmanned Aerial Vehicle (UAV) models have been developed with diverse applications, including maritime traffic monitoring, surveillance, Internet of Things (IoT) communication, mapping, and mobile broadband. Recently, Sceye has successfully launched, flown, and landed its stratospheric airship-type HAPS vehicle, reaching an altitude of 20 km^23^. The company recently conducted tests to demonstrate 100 Mbps download speeds for homes, schools, and clinics. In 2020, Thales Alenia Space and Thales signed an agreement with France’s defense procurement agency for a full-scale, autonomous Stratobus, demonstrating its capabilities to perform a variety of intelligence, surveillance, and reconnaissance missions^24^.

One major benefit of HAPS is the good radio propagation conditions provided by the aircraft’s operational height, which allows for a high probability of line-of-sight with the terrestrial end-user devices, even in the presence of terrain impediments that may otherwise negatively impact terrestrial-based communications. Additionally, HAPS might enhance coverage in coastal areas and link boats at sea that are beyond the range of terrestrial networks. More dependable connectivity along traffic corridors may be possible due to HAPS’ capacity to offer nearly complete geographic coverage with lower latency than satellites. To further reduce the latency for nearly real-time services, a HAPS system can also provide connectivity to the edge computing facilities on the ground.

Recently, machine learning (ML) as a powerful predicting or estimating method has been considered to produce accurate results for a specific task while also having the advantage of learning. The design and optimization processes would be sped up by including an optimizer in the machine learning model since fewer simulations would be needed. With the advancement of neural network science, machine learning for systems of time importance has developed into a fundamental computing tool with high performance and computational efficiency. For instance, these systems encompass steering and beamforming for antenna arrays^25^. Moreover, numerous electromagnetic applications, including multi-input, multi-output (MIMO) systems, antenna design, remote sensing, radar, and direction-of-arrival estimation, leverage these technologies due to their exceptional capabilities^26,27^. A speedy beam-forming synthesis process can be provided by a deep neural network (DNN) while maintaining high accuracy levels, minimizing error and time savings, and possibly predicting the antenna behavior. DNNs also have improved computing efficiency and require fewer simulations. As a result, the suggested DNN model provides a precise and resilient computing technique that may be used instead of costly measurement and simulation. Refs^26,27^. presents a comprehensive assessment of several research publications on the development and optimization of antennas by machine learning, covering many methodologies and algorithms used to generate antenna parameters based on required radiation patterns and other antenna requirements. The authors of^28^ synthesized the radiation patterns using an array of 41 patch antennas resonating at 2.4 GHz with an inter-element spacing of 0.28λ. The radiation pattern was used as the input, while the phase and amplitude of the antenna elements were used as the output. The suggested DNN has indeed been trained on a large variety of radiation modeling data and is capable of generating radiation patterns. The ability to create radiation patterns with certainty using deep learning was thus demonstrated. Because of the inherent nonlinearities in their emission patterns, antennas are considered suitable candidates for DNNs in general. DNN is used in^29^ to softly calculate a dual-band circularly polarized CP bone-shaped patch antenna for fifth-generation applications at 28 GHz and 38 GHz. The main beam pattern of the designed uniform circular antenna array with a side-lobe level (SLL) less than − 30 dB is then beam-steered using DNN with back-propagation (BP) technique and the weighted modified gravitational search algorithm-particle swarm optimization (MGSA-PSO) algorithm by assessing the suitable exciting phases of the 16 elements. The same approach is used in^30^ to steer the beam pattern of a 64-hybrid plasmonic nano-antenna array operating at 1550 nm. In^31^, the DNN is employed to design an intelligent reflector metasurface for 6G adaptive beamforming. This approach demonstrates the ability to accurately learn and predict the parameters influencing the reflected wave radiation pattern, achieving accuracy comparable to full-wave simulations (98.8–99.8%) while significantly reducing computational time and complexity compared to analytical methods. Furthermore, in^32^, a multi-resonance applicator (MRA) is designed for health applications using a combination of machine learning and the MGSA-PSO algorithm. Comparative analysis with a single-resonance applicator (SRA) reveals that the MRA exhibits superior performance and reduces processing time by up to 42.5%.

For beamforming applications, a phase shifter is needed for each element in the array to generate different phases. There are different types of phase shifters, such as switched-line, loaded-line, reflection-type, and Microelectromechanical systems (MEMS)^33^. Each type has its advantages and disadvantages in terms of performance, complexity, cost, and size. The choice of phase shifter depends on the frequency, bandwidth, power, and cost requirements of the application. Most phased array antennas that have been designed in the past few years have used analog beamforming, where the phase adjustment is done at radio frequency or intermediate frequency frequencies and there is one set of data converters for the entire antenna. There is increased interest in digital beamforming, where there is one set of data converters per antenna element and the phase adjustment is done digitally in the Field Programmable Gate Array (FPGA) or some data converters. There are many benefits to digital beamforming starting with the ability to transmit many beams easily or even change the number of beams almost instantly. There are multiple considerations to make when considering analog vs. digital beamforming. A common approach to beamforming involves a hybrid architecture, combining analog and digital beamforming techniques. This approach typically utilizes subarrays for analog beamforming, followed by digital signal processing to combine the signals from these subarrays. This is an area of growing interest in the industry and will continue to evolve in the years to come^34,35^. In^36^, the circular interval analysis is used to derive analytic and reliable power pattern bounds used within a convex programming-based optimization, such that the lower bound of the power pattern main beam peak is maximized while the sidelobes are constrained by an arbitrary upper-bound mask. In^37^, a programmable beam-scanning antenna without phase shifters operating in the X-band is designed to evaluate the feasibility of a new type of antenna design scheme that can be used for IoT relay communication. The integrated design of the excitation source and phase-modulated structure greatly reduces the profile of the antenna and improves the integrated degrees of freedom with other equipment.

Nowadays, HAPs have captured a great deal of attention as a leading candidate for coverage extension or the need for more broadband connectivity. Whereas, it may be difficult to provide terrestrial networks in rural locations due to network economics and planning restrictions. There may be frequent connectivity pauses owing to poor service, as many people discover when traveling by train, automobile, or bus. However, commuters and other travelers, as well as self-driving cars and trucks, need reliable, all-encompassing service. Coverage issues are typically caused by the terrain’s shape, as hills and other geographical characteristics block signals from nearby terrestrial stations. As a result, certain regions have equally bad coverage for all carriers. These “white spots” are frequently small, irregular areas; thus, covering them would necessitate the deployment of numerous new terrestrial sites, which is not possible from an economic standpoint^17^. In contrast, HAPS are very versatile; they can be adjusted to prioritize coverage or capacity depending on the use case. Generally, the coverage achieved by a conventional HAP station is limited to a circular area with a radius of tens of kilometers. However, in some cases, it is required to cover only the roadway or railway route located in rural or rugged terrain areas without the need to cover the surrounding zone. Recently, in^38^, SoftBank and HAPS Mobile researchers have presented a cylindrical massive MIMO system for HAPS communications operating at 2.5 GHz that can create multiple high-gain beams. However, this paper proposes a dynamical coverage of a given path by synthesizing multiple vertical/horizontal (V/H) directional beams to overcome the conventional HAP station, which can be summarized in the following points focusing on conciseness and clarity:

- Efficient Area Coverage: Prioritize coverage of designated areas (e.g., roads within defined boundaries) while minimizing power dissipation in unpopulated regions.
- Enhanced Interference Mitigation: Simultaneous coverage with both Vertical (V-pol) and Horizontal (H-pol) beams can reduce interference between adjacent zones and improve frequency reuse efficiency.
- Improved Signal Quality: Concentrate radiated power along the desired road path, leading to increased signal strength, improved Signal-to-Interference-Plus-Noise Ratio (SINR), and enhanced system capacity.
- Dynamic Beam Steering: Utilize a DNN to synthesize multiple V/H directional beams, enabling the system to dynamically cover any road path by adjusting the feeding phases of the antenna elements.
- Adaptive Beam Steering: Employ the DNN to enable the HAPS station to adapt beam directions in real-time based on changes in the road path or the HAPS’ position.
- Dynamic Footprint Correction: Utilize the DNN to adaptively correct the ground footprint by adjusting beam directions in response to changes in the HAPS position or variations in the road path.

This paper presents a Concentric Circular Array (CCA) with 60 antenna elements resonating at 2.1 GHz for dynamical coverage of a given path by synthesizing multiple vertical/horizontal (V/H) directional beams. So, the DNN-based model is optimized in the training phase of a hybrid MGSA-PSO algorithm for synthesizing multiple V/H directional beams afforded to the road path to be covered. Whereas the beam patterns will be generated according to the path of the covered area. In such a case, ML-based beamforming at the HAPS station is introduced. Firstly, by predicting the suitable feeding phases, a DNN with a BP method and a weighted MGSA-PSO algorithm are employed for the multi-beamforming pattern of a CCA with 60 antenna elements. The key challenge is funding optimal CCA element weights that result in a beamforming radiation pattern in directions capable of covering the intended area. To verify the technique’s validity, several illustrative examples are placed to beamform the pattern toward the desired covered area. We can summarize the novelty in this paper at the following points:

- A novel single-antenna element, designed to serve as a building block for an antenna array, was optimized using the MGSA-PSO algorithm. This optimization aimed to minimize reflection and maximize antenna gain within the desired operating frequency band. Notably, the antenna is capable of propagating dual polarization modes.
- The design of CCA incorporates 60 elements and possesses the capability to synthesize multiple V/H (Vertical/Horizontal) directional beams.
- Environmental test models were simulated by combining the Computer Simulation Technology - Microwave Studio Suite (CST-MWS) program with the Earth Explorer (EE) user interface tool.
- This research proposes the application of a DNN with a BP algorithm and a weighted MGSA-PSO algorithm to estimate the optimal feeding phases for CCA elements. This enables the generation of multi-V/H beamforming patterns that can effectively cover different road paths.
- Considering various road types and paths, including terrain roads and rural roads, the proposed technique demonstrates the ability to efficiently cover the entire length of the road.

This paper is organized as follows: Section II presents the antenna design and CCA configuration. Section III provides a brief overview of the DNN. Section IV presents the results and discussion. Finally, Section V concludes the paper.

## Design considerations and simulation methodology

In this section, the proposed antenna element’s design structure and the 60 antenna elements’ geometrical configuration study are described.

### Configuration of CCA design

**Fig. 1:**
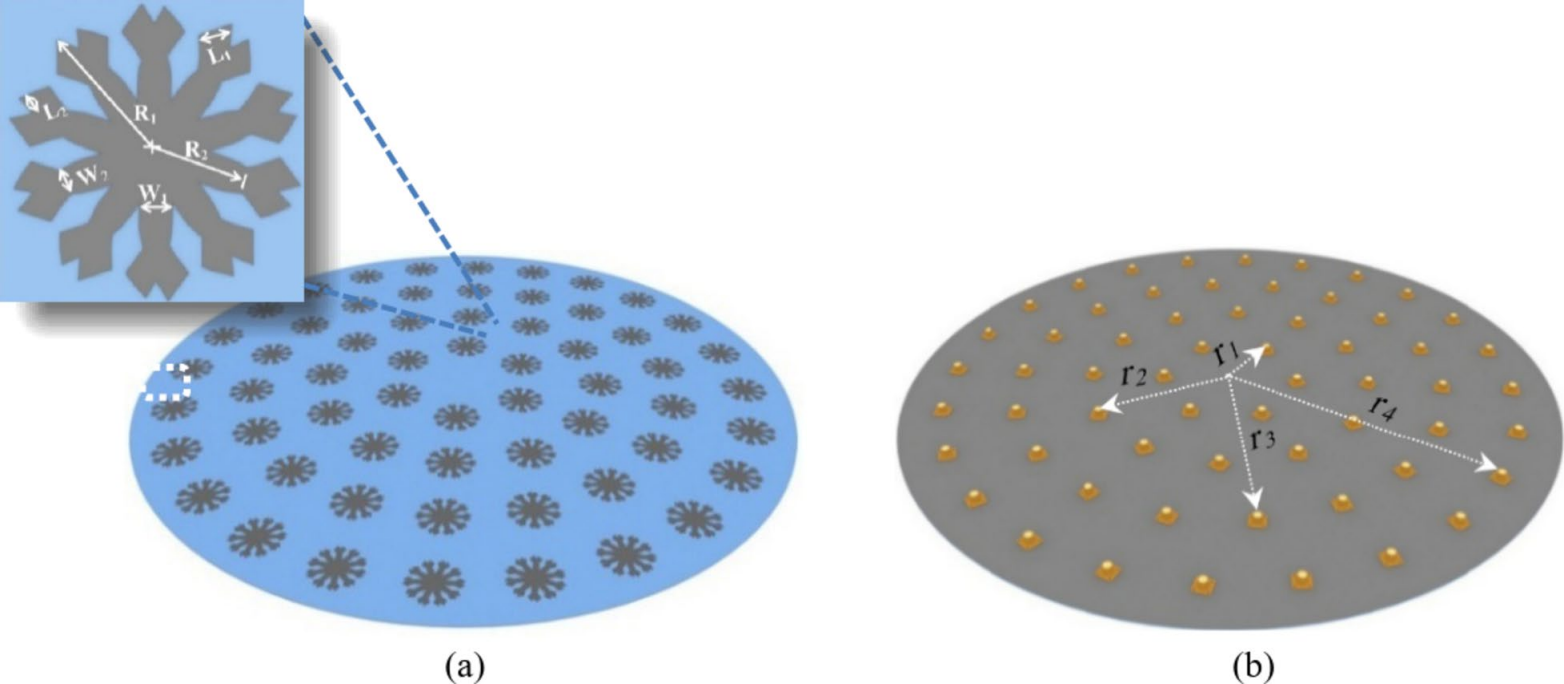
Proposed CCA structure, (**a**) Front view, (**b**) Back view. The inset figure illustrates the one-element dimensions.

Generally, the CCA array has many benefits, including flexibility in array pattern synthesis and design both in narrowband and broadband beam-forming applications, especially at the base stations, as in our case. The suggested CCA is depicted in Fig. 1. The number of antenna array elements is computed based on the constraint of the assigned array gain, which is assumed to be greater than 25 dBi. Generally, the maximum gain that can be obtained for N-antenna elements is equal to the one-element gain plus 10 log10 (N). In our case, the antenna gain element is found to be 7.68 dBi; therefore, 60 elements are needed to obtain an antenna array gain of 25 dBi. As the number of elements increases, higher antenna array gain can be obtained, but at the expense of complexity and cost. To minimize the mutual coupling effect between the CCA antenna elements, a separation distance between antenna elements in all directions is considered to be 0.5λ.

The CCA geometry is proposed to consist of four concentrically arranged circular arrays with regularly spaced antenna elements. The total number of the CCA is distributed through the four rings, where the number of elements in each ring is $\:6\times\:i$, and $\:i$ is the ring number. Therefore, the inner ring of radius r_1_ = 1.5λ (the distance between the center of the CCA and the antenna feeding point) consists of six elements. Consequently, the second ring contains 12 elements distributed uniformly along the circumference of radius r_2_ = 3λ. Thus, the third ring of radius r_3_ = 4.5λ contains 18 elements. Lastly, the fourth ring contains 24 elements disseminated along the ring circumference of radius r_4_ = 6λ. The CCA is designed on a substrate radius of 7λ, where λ is the wavelength of 2.1 GHz.

Each antenna element is fed with 50 ohms of coaxial cable in the center of the antenna. The antenna element consists of a circular disc from which ten similar beams come out, with an angle of 36^o^ between each beam and the other. Each ray at its end splits into two small paths, as shown in the inset of Fig. 1. Each part of the antenna has a specific importance. For example, the ray radius R_2_ without splinter paths directly affects the antenna reflection coefficient and correspondingly the antenna matching, the overall antenna radius R_1_ directly affects the antenna realized gain, the width of the ray parameter W_1_ directly affects the antenna radiation efficiency, and finally, the splinter ray length L_2_ directly affects the antenna’s polarization and its axial ratio. The antenna will be fabricated on the FR-4 substrate, which is cheap and easily available with properties such as relative permittivity $\:{{\upepsilon\:}}_{\text{r}}$ = 4.4, loss tangent $\:\text{t}\text{a}\text{n}{\updelta\:}\hspace{0.17em}$= 0.02 and substrate thickness = 1.6 mm. The proposed CCA is built to combine many beam patterns in various directions. By varying the input signal phase assigned to each antenna element, it is possible to steer the radiation pattern with a high gain and a side-lobe level of less than − 30 dB. The MGSA-PSO algorithm is proposed to optimize the feeding phases.

### Environmental test models

**Fig. 2:**
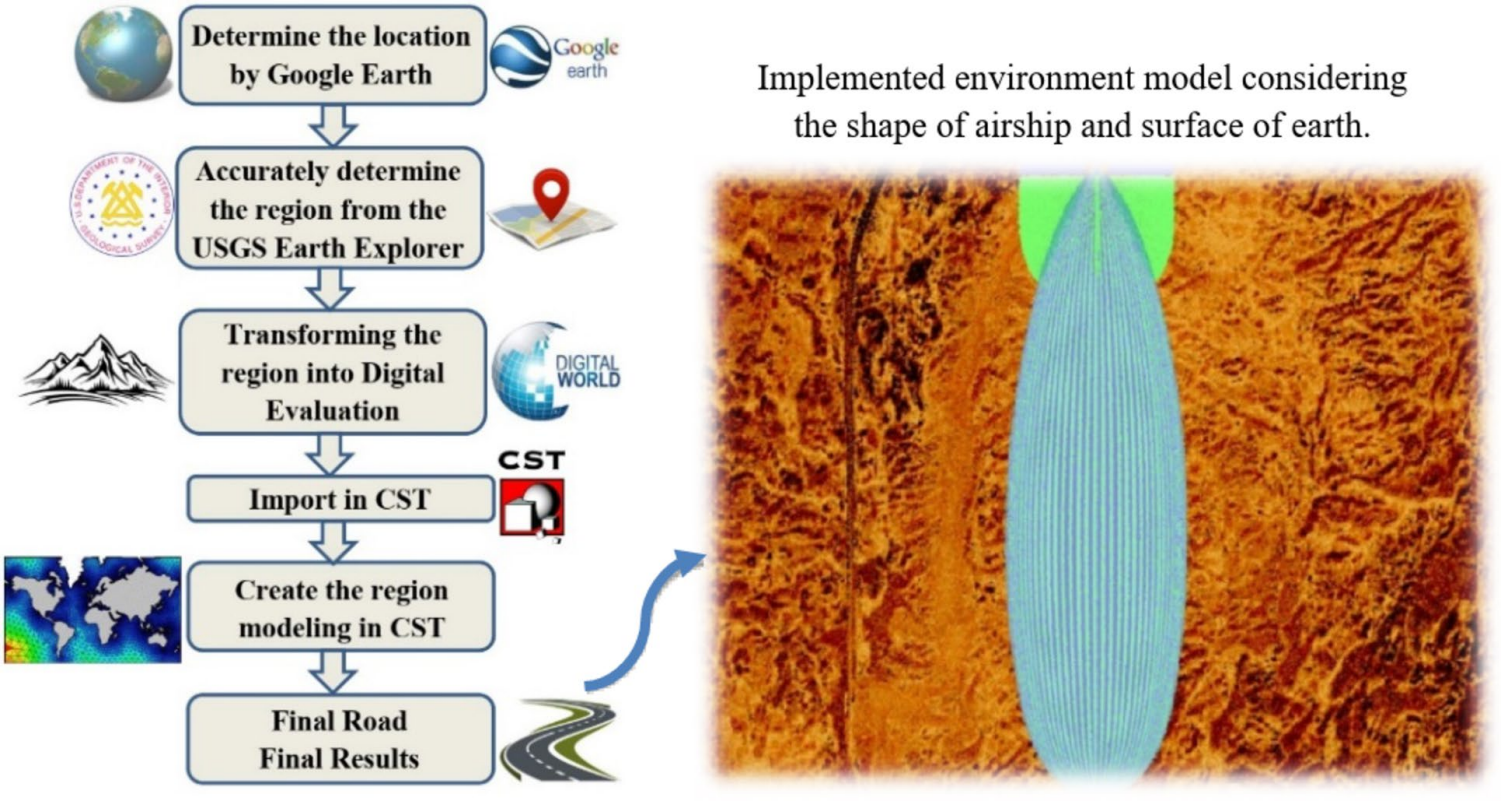
The main steps to simulate the road on the CST-MWS program. The inset figure illustrates the implemented environment model considering the airship shape and earth surface.

In this work, the performance of the proposed CCA will be examined to cover crooked paths with different terrains using DNN. To create a road on the CST-MWS program that simulates the real road that exists in reality, the following steps are accomplished, as depicted in Fig. 2.

- Step 1: Determine the area that needs to be covered by assigning its longitude and latitude to the Google Earth program.
- Step 2: Determining the coordinates of the boundaries of the region from all directions with the Earth Explorer (EE) user interface tool developed by the United States Geological Survey (USGS)^39^, which provides multiple extensions for different types of maps.
- Step 3: Choose the digital elevation form to export the image of the assigned area with the values of highs and lows above sea level.
- Step 4: Importing the digital image into the CST-MWS program and then designing a plane with high meshes. We form this plane on the surface of the specified area with its highs and lows and corresponding medium electric properties.
- Step 5: Finally, the CCA is integrated with an air balloon (an airship) to simulate reality. Whereas, the material of the airship is rubber with εr = 2.5 and a loss tangent of 0.005. The CCA is placed facing down at an altitude of 22 km above sea level, as shown in the insFig.  Fig. 2.

**Fig. 3:**
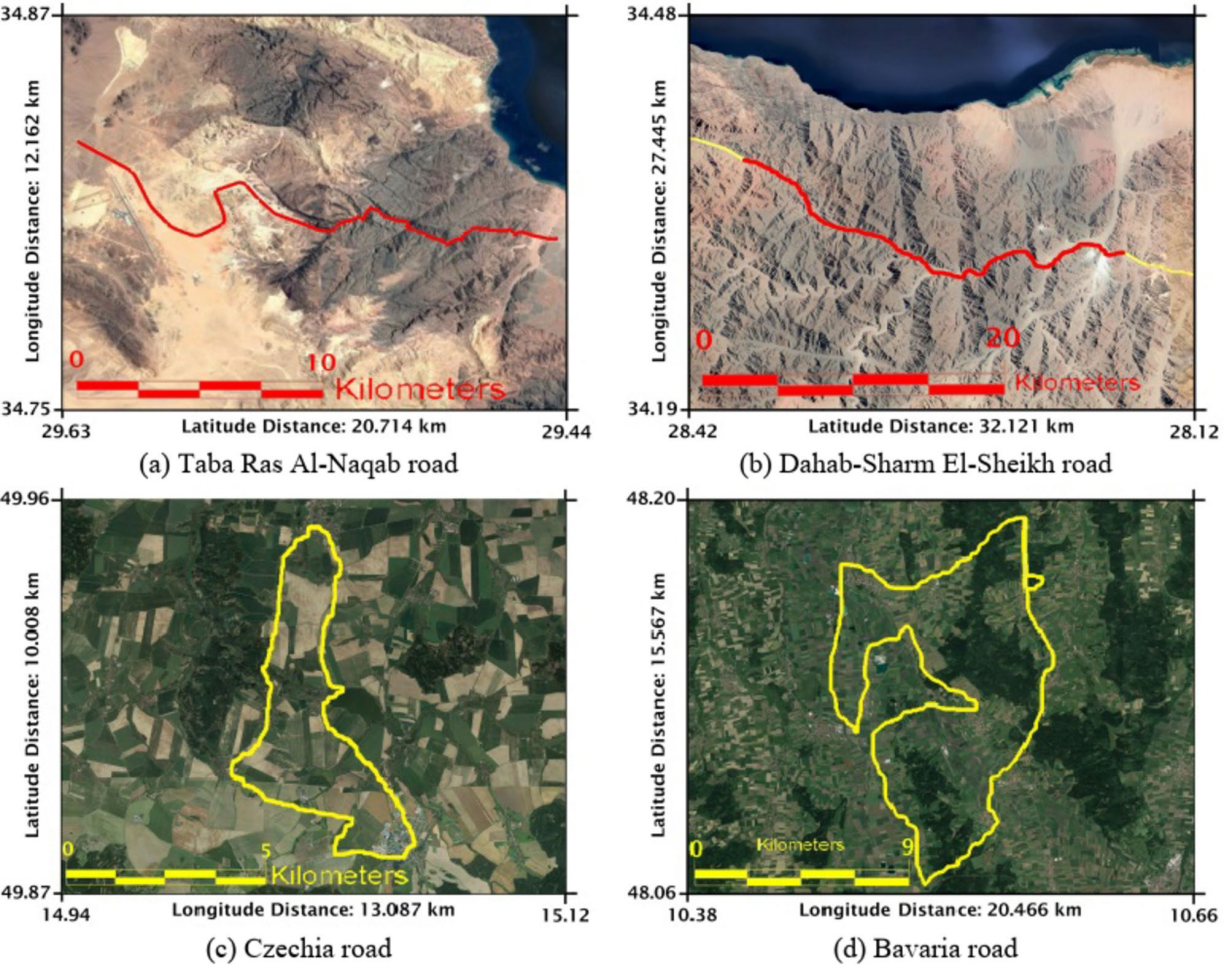
The map and road of different crooked paths with different terrains.

With this technique, many roads with different environments were considered and implemented, as shown in Fig. 3, including the rugged desert areas with mountain chains, as in the example of the Taba Ras Al-Naqab and Dahab-Sharm El-Sheikh roads in Egypt. In addition, other roads, such as those in Czechia and Bavaria in Germany, are considered areas where forests and trees abound.

**Fig. 4:**
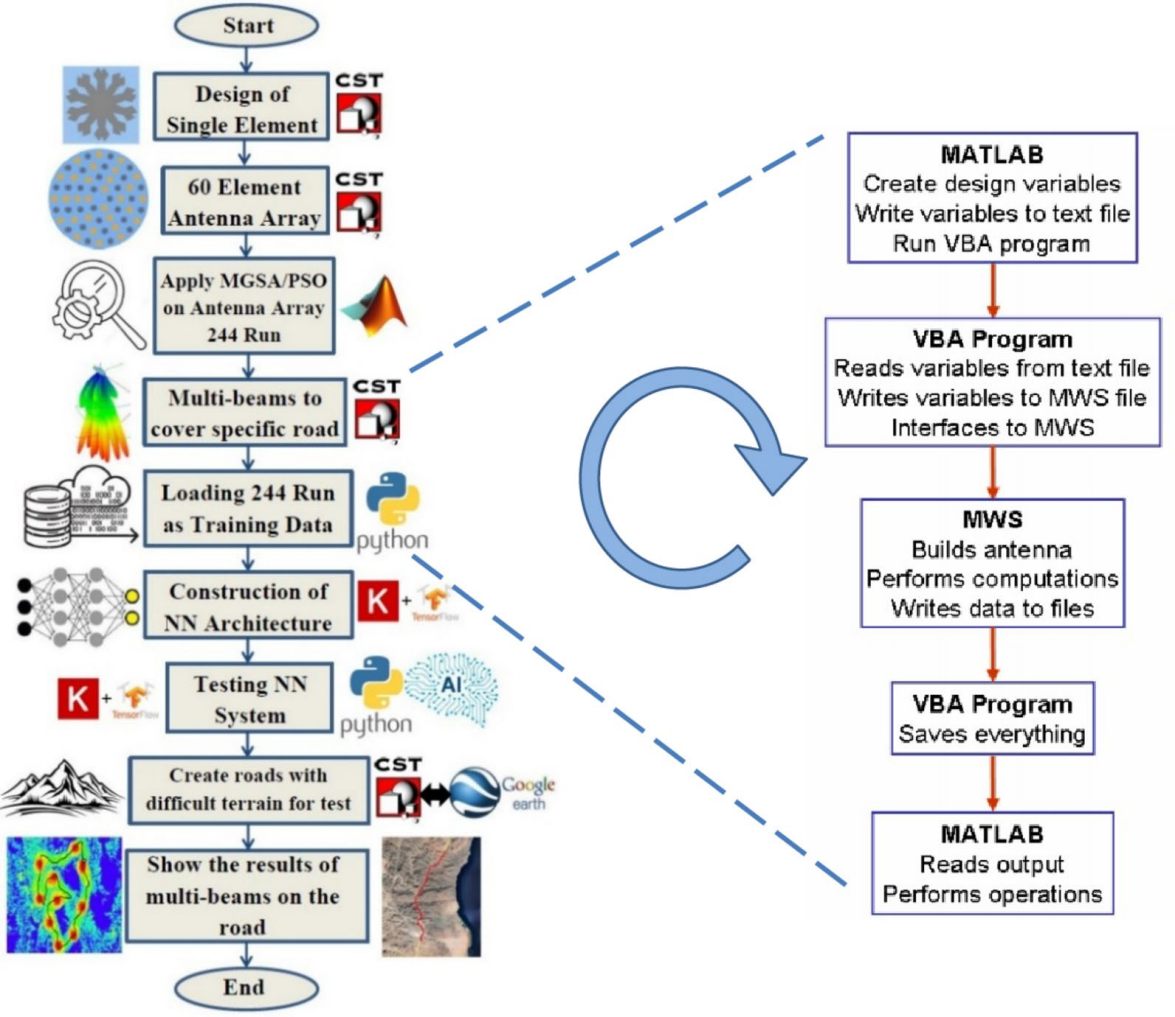
The main steps of the design process of the DNN-based approach for HAPS multi-beams beamforming design including the flow chart for Matlab/CST-MWS Interfacing.

It should be noted that each scenario has different incident angles and coordinates, representing different cities, maps, and regions with variable terrain within the HAPS coverage, which eventually resulted in an advanced predictive system that can cover any area on the map with high accuracy and quality. To prevent interference between the two adjacent covered cells, the adjacent cells are covered with different polarity beams simultaneously; one of them is E_θ_ and the other is E_φ_, respectively. In this target, Fig. 4 shows the methodology used in this research for multi-beam beamforming design based on the DNN approach. The main steps of the proposed technique start with the design of the single-element antenna to have the ability to achieve high gain and circular polarity. Then, a 60-element CCA antenna array was designed. Applying a powerful algorithm and high-precision optimization technique, MGSA-PSO, which works on beamforming multi-beams in certain places to cover a specific road or an area, each of which has a different polarity from the polarity of the next spot so that there is no interference between the beams and each other, we cover N different roads, including horizontal roads, vertical roads, tile to the right, and tile to the left, in four different groups. These N coverage places are considered a database of training data. To meet the requirement for creating a powerful design and system analysis environment, Matlab is used to control commercial electromagnetic software such as CST-MWS. The interfacing between Matlab and CST-MWS is created via a scripting language called Visual Basic for Applications (VBA script)^40,41^. A VBA script incorporates a set of instructions written in a scripting language in the form of a text file. Interfacing steps between Matlab and CST-MWS are shown in the inset of Fig. 4. With each run in the microwave studio, a huge number of files are generated. After invoking CST, the VBA program reads the data file generated by Matlab. The MWS file is opened once the data is read. When the scripting language opens the file, the commands in the script are executed. The MWS window closes automatically, and the control returns to Matlab using the release command when the solver finishes and stores the data. Whenever the program is called from Matlab, the microwave studio window will reopen.

This training data was entered into Python to design the structure of the neural network architecture for the DNN system. After training the DNN system well with the previous data, we evaluate this system by covering places and roads that the system has not previously trained. At the end, the obtained results are introduced, along with the percentage of error in the results, indicating the approach’s capability of adapting to any road or place to be covered, regardless of the difficulty of the surrounding terrain.

### Modified GSA-PSO algorithm

Global optimization techniques are powerful tools for array pattern synthesis, effectively navigating the search space to identify global or near-global optima while avoiding local minima. A variety of stochastic global optimization methods have been successfully applied to this problem, including Genetic Algorithms (GA)^42^, Bee Algorithms^43^, Simulated Annealing (SA)^44^, Particle Swarm Optimization (PSO)^45^, Ant Colony Optimization (ACO)^46^, Central Force Optimization (CFO)^47^, and Gravitational Search Algorithms (GSA)^48^. Furthermore, hybrid approaches, such as the combination of GSA and PSO (GSA-PSO)^49^, have been proposed to leverage the strengths of both constituent algorithms.

The Modified GSA-PSO algorithm offers several key advantages in the context of antenna array beamforming. By synergistically combining the global exploration capabilities of the Gravitational Search Algorithm (GSA) with the efficient exploitation characteristics of Particle Swarm Optimization (PSO), the algorithm effectively navigates the complex optimization landscape. This hybrid approach enhances the ability to locate optimal solutions while mitigating the risk of premature convergence to suboptimal local minima. Furthermore, the incorporation of dynamic threshold optimization further refines the search process, accelerating convergence and improving robustness in handling the challenging, multi-modal optimization problems frequently encountered in antenna array design. This combination of features makes the Modified GSA-PSO a highly effective tool for optimizing beamforming systems, particularly in scenarios where achieving optimal performance necessitates navigating a complex and challenging search space.

Therefore, this paper considers the modified version of the hybrid gravitational search algorithm GSA and particle swarm optimization PSO with dynamic threshold optimization (DTO) search capability is considered due to its high convergence performance. In MGSA-PSO, the position of each agent $\:{X}_{i}\:$provides a solution to the optimized problem, whereas the mass value $\:{M}_{i}\left(t\right)\:$of each agent $\:i$ is determined according to its fitness value $\:{fit}_{i}\left(t\right)$.

1 $$\:{M}_{i}\left(t\right)=\frac{{q}_{i}\left(t\right)}{\sum\:_{j=1}^{S}{q}_{j}\left(t\right)}\:\:\:,$$

2 $$\:{q}_{i}\left(t\right)=\frac{{fit}_{i}\left(t\right)-\:worst\left(t\right)}{best\left(t\right)-worst\left(t\right)}\:\:,$$

where $\:{fit}_{i}\left(t\right)$ represent the fitness value of agent $\:i$ at iteration $\:t$, $\:Np$ is the population size. $\:best\left(t\right)\:\:$and $\:worst\left(t\right)$are defined as follows:

3 $$\:{best}\:\left(t\right)={Max}{fit}_{j}\left(t\right)\:\:\:\:\:\:j\in\:\left\{1,\:\dots\:,\:m\right\}.$$

4 $$\:{worst}\:\left(t\right)={Min}{fit}_{j}\left(t\right)\:\:\:\:j\in\:\left\{1,\:\dots\:,\:m\right\}.$$

To compute the agent acceleration, the total forces from a set of heavier masses applied to it should be considered based on the gravity law, which is followed by the law of motion as follows:

5 $$\:{a}_{i}^{d}\left(t\right)=\frac{F{}_{i}{}^{d}\left(t\right)}{{M}_{i}\left(t\right)}=\sum\:_{j\ne\:i}{rand}_{j\:}G\left(t\right)\frac{{M}_{j}\left(t\right)}{{R}_{ij}\left(t\right)+\epsilon\:}\left({x}_{j}^{d}\left(t\right)-{x}_{i}^{d}\left(t\right)\right)\:\:\:\:\:\:\:\:\:\:\:\:\:\:\:\:\:\:\:\:\:\:\:\:\:\:$$

Where $\:{\:R}_{ij}\left(t\right)$is the Euclidean distance between two agents, $\:i$ and $\:j$, $\:\epsilon\:$ is a small value. $\:G\left(t\right)$ is the gravitational constant which is decreased exponentially with time $\:t$ as follows:

6 $$\:G\left(t\right){=G}_{0}exp(-\beta\:t/{t}_{max})$$

where $\:{G}_{0}$ is the initial value, $\:\beta\:$ is a constant, and $\:{t}_{max}$ is the maximum number of iterations.

In the modified version of the GSA-PSO algorithm, the time-varying inertia weight $\:\omega\:\left(t\right)$ in addition to the time-varying acceleration coefficients $\:{c}_{1}\left(t\right)$ and $\:{c}_{2}\left(t\right)$ are proposed to effectively control the global search and convergence towards the global best solution^49^. Therefore, the next velocity of an agent $\:i$,$\:\:{v}_{i}^{d}\left(t+1\right)$, is calculated based on a fraction of its current velocity added to the ability of social thinking ($\:gbest$) in the PSO and its acceleration, as in Eq. (7). Then, its next position,$\:\:{x}_{i}^{d}\left(t+1\right)$, can be calculated using the following equations, respectively:

7 $$\:{v}_{i}^{d}\left(t+1\right)=\omega\:\left(t\right){v}_{i}^{d}\left(t\right)+\:{{c}_{1}\left(t\right)\:{rand}_{i}\:a}_{i}^{d}\left(t\right)+{c}_{2}\left(t\right)\:{rand}_{i}\left(gbest-{x}_{i}^{d}\left(t\right)\right)\:\:\:\:\:\:\:\:\:\:\:\:\:\:\:\:\:$$

8 $$\:{x}_{i}^{d}\left(t+1\right)={x}_{i}^{d}\left(t\right)+\:{v}_{i}^{d}\left(t+1\right)\varDelta\:t\:\:\:\:\:\:\:\:\:\:\:\:\:\:\:\:\:\:\:\:\:\:\:\:\:\:\:\:\:\:\:\:\:\:\:\:\:\:\:\:\:\:\:\:\:\:\:\:\:\:\:$$

where, $\:{rand}_{i}$ and $\:{rand}_{j\:}$ are random numbers in the interval [0, 1] to give a randomised characteristic to the search.$\:\:gbest$ is the best solution so far and $\:\varDelta\:t$ is the unit time step. In each iteration $\:t$, the current weighting value and acceleration coefficients can be calculated using the following equations:

9 $$\:\omega\:\left(t\right)={\omega\:}^{max}-\frac{t}{{N}_{t}}({\omega\:}^{max}-{\omega\:}^{min})\:\:\:\:\:\:\:\:\:\:\:\:\:\:\:\:\:\:\:\:\:\:\:\:\:$$

10 $$\:{c}_{1}\left(t\right)={{c}_{1}}^{max}-\frac{t}{{N}_{t}}({{c}_{1}}^{max}-{{c}_{1}}^{min})\:\:\:\:\:\:\:\:\:\:\:\:\:\:\:\:\:\:\:\:\:\:$$

11 $$\:{c}_{2}\left(t\right)={{c}_{2}}^{min}+\frac{t}{{N}_{t}}({{c}_{2}}^{max}-{{c}_{2}}^{min})\:\:\:\:\:\:\:\:\:\:\:\:\:\:\:\:\:\:\:\:\:\:\:$$

By updating$\:\:\omega\:$, $\:{c}_{1}$ and $\:{c}_{2}$ as depicted, the global and local search abilities can be balanced. With a large cognitive component and a small social component at the beginning, the probes are allowed to move around the search space, instead of moving toward the population best. However, a small cognitive component and a large social component allow the probes to converge to the global optima in the later part of the optimization.

**Fig. 5:**
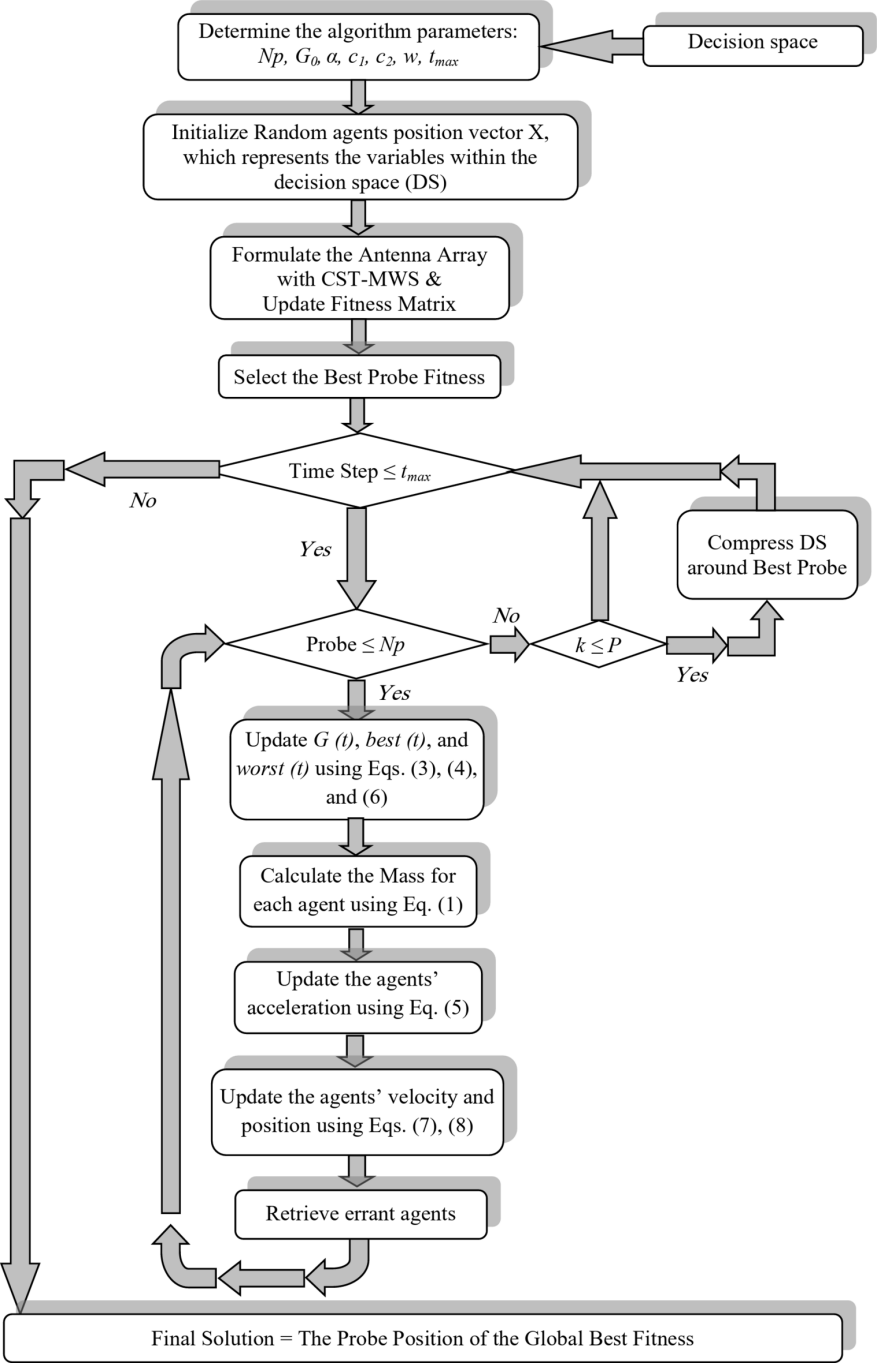
Flowchart showing the main steps of the MGSA-PSO algorithm.

Furthermore, the decision space (DS) in the MGSA-PSO is adaptively compressed by the dynamic threshold optimization (DTO) that was introduced in^50^. This approach is based on compressing DS from below in the direction of the dependent variable using a series of successively increasing “thresholds” instead of shrinking DS. Figure 5 shows the general process flowchart for the creation and implementation of the MGSA-PSO algorithm/simulation interface, while Table 1 shows the values of the MGSA-PSO coefficients used in this work. The MGSA-PSO algorithm as a powerful optimization tool will be considered to optimize the design of one antenna element for high gain, matching input impedance, and high efficiency. Then, the algorithm is considered to control the antenna array phase’s excitations to cover a certain road to use the data as learning inputs to DNN.

**Table 1:** The values of the MGSA-PSO coefficients.

Algorithm coefficient	$\:{\upalpha\:}$	$\:{\text{G}}_{0}$	$\:{{\upomega\:}}^{\text{m}\text{a}\text{x}}$	$\:{{\upomega\:}}^{\text{m}\text{i}\text{n}}$	$\:{{\text{c}}_{1}}^{\text{m}\text{a}\text{x}}$	$\:{{\text{c}}_{1}}^{\text{m}\text{i}\text{n}}$	$\:{{\text{c}}_{2}}^{\text{m}\text{a}\text{x}}$	$\:{{\text{c}}_{2}}^{\text{m}\text{i}\text{n}}$
Value	20	1	0.9	0.4	1.5	0.5	1.5	0.5

The Modified GSA-PSO algorithm, while offering significant advantages in optimizing beamforming solutions, presents certain computational considerations. The training phase exhibits a time complexity of O(3 *N* + 3ND), where N represents the number of particles and D represents the dimension of the decision space, influenced by the DNN model’s complexity and the number of antenna elements. This phase demands substantial computational resources, including powerful Central Processing Units (CPUs) and Graphics Processing Units (GPUs), particularly when dealing with larger particle swarms and complex DNN architectures. Significant memory is also required to store particle parameters, the DNN model, and training data. While the deployment phase necessitates fewer computational resources compared to training, it still requires sufficient processing power for real-time operation and adequate memory on the HAPS platform to store the DNN model and its associated parameters. The DNN model’s computational complexity is primarily determined by its architectural characteristics, such as the number of layers and neurons.

The MGSA-PSO algorithm has already been presented and tested by the authors in different applications^49,51–54^, achieving a better competence to escape from local optima than the classical version of GSA-PSO. It is found that the MGSA-PSO algorithm outperformed the classical GSA-PSO algorithms by 22.2%. Furthermore, the modified GSA-PSO algorithm enhanced the convergence capability by 15% compared with the GSA-PSO algorithm^49^.

### Deep neural network (DNN)

Processing a large number of neurons instantaneously on several layers, capturing features from them, and evaluating optimal network parameters through regression/classification are all advantages that distinguish deep learning from machine learning. It also has a hierarchy of neurons where data is evaluated and moved through this hierarchy to the higher layers, thus retaining important and improved features in the higher neurons and allowing for a more appropriate learning model to be built.

For establishing the training data, the CCA, which consists of 60 elements, is designed to beam-form the pattern in up to 10 different desired directions in the same scenario at the same time with as high a gain and as low an SLL as possible. Therefore, a high-efficiency and powerful MGSA-PSO algorithm is used to achieve the goal by controlling the phase feeding of each antenna element of the CCA. Whereas a set of agents is randomly generated at the start of the MGSA-PSO optimization process. Each agent represents the phases of the CCA elements (ϕ_1_-ϕ_60_) and is a possible solution to the beam-forming optimization problem. New generations of agents will be obtained according to the algorithm’s methodology depicted in^29–32,49,51–54^. This scenario has been achieved when applying the algorithm MGSA-PSO to the phase feed in the CCA using the following objective function:

12 $$\:{\text{O}\text{b}\text{j}}_{1}\:=\:\sum\:_{\text{i}=1}^{\text{N}}\left|{\text{E}}_{{\uptheta\:}}\left({{\uptheta\:}}_{\text{i}},{{\upphi\:}}_{\text{i}}\right)\right|+\sum\:_{\text{j}=1}^{\text{M}}\left|{\text{E}}_{{\upphi\:}}\left({{\uptheta\:}}_{\text{i}},{{\upphi\:}}_{\text{i}}\right)\right|$$

where E_φ_ and E_θ_ are the horizontal and vertical field intensities, respectively. The constant N denotes the number of desired angles that will receive V-pol. signals, whereas M symbolizes the number of desired angles that will be covered with H-pol. signals. This Obj. function was written to consider various possible scenarios, whereas the array can direct V/H multi-beam patterns adopting directivity and polarization control^49,51^. In some scenarios, it may be necessary to cover the spot with circular polarization waves. In this case, the V-beams and H-beams should be directed in the same direction; in addition, the amplitudes of the vertical and horizontal components in these directions should be approximately equal^54^. In this paper, the main objective is to be able to cover the roadway with a V/H multi-beam pattern simultaneously to limit interference considering the same number of V-beams and H-beams (N = M = 5). In general, the number of V-beams may be different from the number of H-beams. Also, the directions of V-beams may differ for H-beams. The machine-learning neural networks will use this data as a training set.

**Fig. 6:**
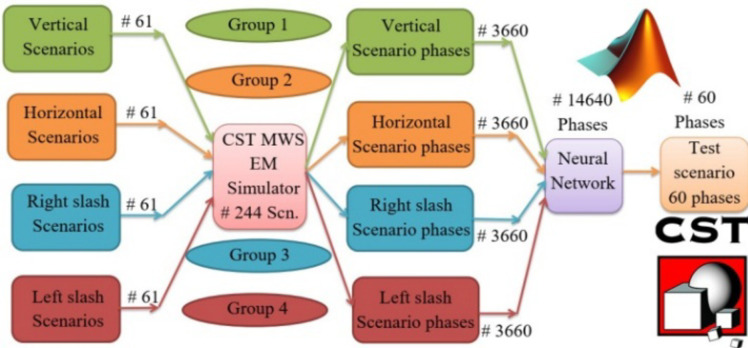
Topological illustration of the four groups that make up the neural network system.

Figure 6 illustrates the topological construction of the DNN system. It is crucial to identify the types of inputs and outputs because these factors are wholly responsible for the learning outcomes. In this study, the nature of the data entered into the DNN is 244 scenarios, and each of these scenarios generates beamforming at specific and different angles in the horizontal or vertical direction. The introduced scenarios were divided into four groups according to the CCA coverage of the angles in the scenarios, as shown in Fig. 6. The first and second groups are called the vertical and horizontal scenarios, respectively, whereas the angles to be covered are in one vertical or horizontal plane. For both, phi or theta angles are ranged from − 30^o^ to 30^o^ with a step of 1^o^ which results in 61 scenarios in the entire group. While the third and fourth groups, which are called the right-slash and left-slash groups, aim to train the DNN on the 61 right-slant and left-slant angles, respectively. As noted, each group contains 61 scenarios, which results in 244 scenarios as the total number of scenarios needed to train the network. Of course, each of these scenarios was implemented through the CST-MWS package and the MGSA-PSO algorithm to control the beam-forming pattern according to the road path (desired θ and φ angles to be covered). The MGSA-PSO algorithm sets the 60 feeding phase values (phase for each element) for each scenario. Thus, the number of phases determined by the MGSA-PSO algorithm in one group is 3660. So, the total number of angles for the DNN as a whole is 3660 × 4 = 14,640 phases. From there, the DNN has been trained to produce any test scenario that is not included in the training scenarios.

**Fig. 7:**
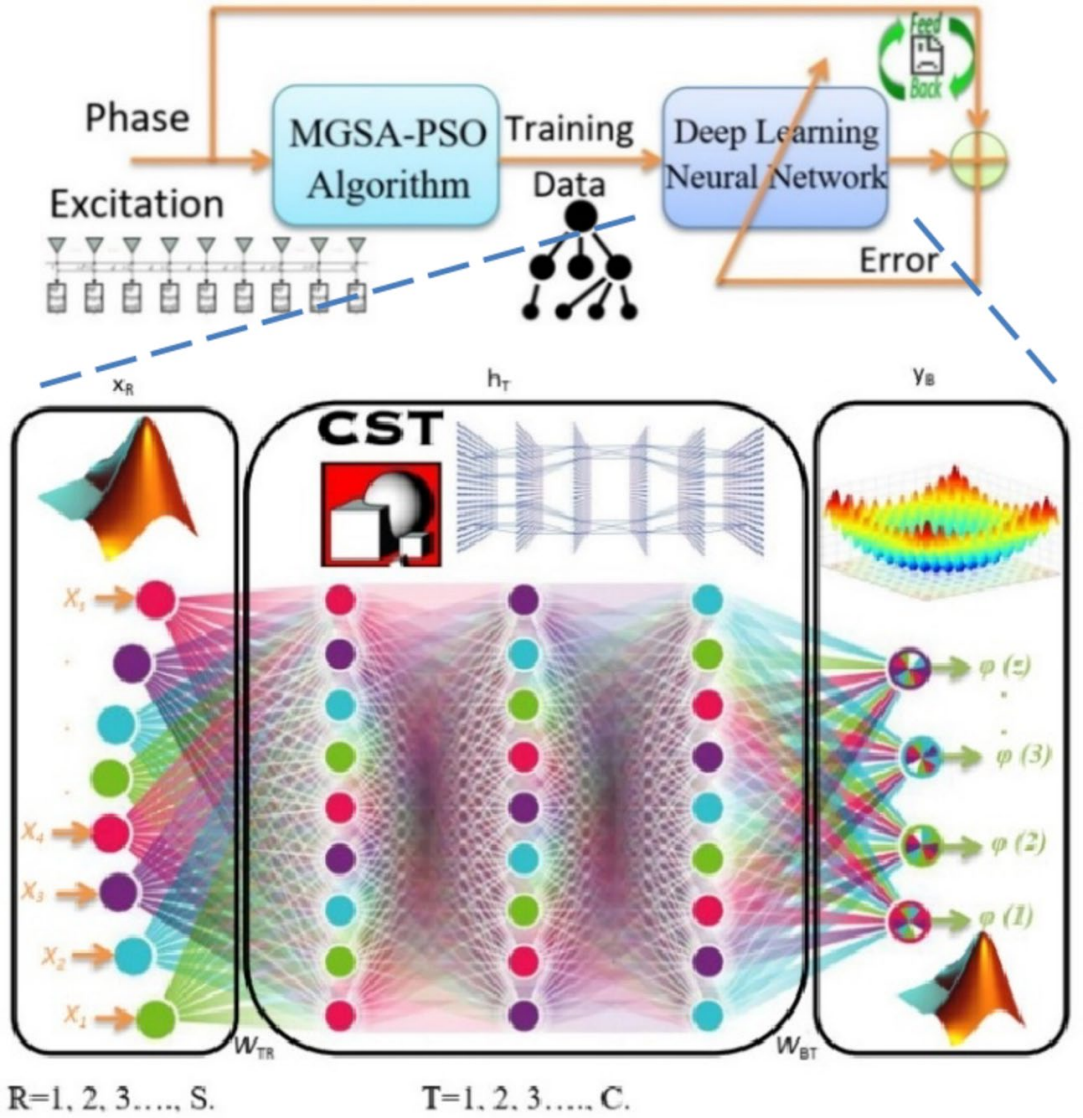
Neural network training procedure including the neural beam-former architecture.

As shown in Fig. 7, the DNN consists of multiple layers. The input layer is considered a support for information in addition to the output layer, and hidden internal layers with variable reconfiguration. Each layer of the network has a specific structure and purpose, so the neurons in one layer do not communicate with each other. The first layer is the input layer, and the number of nodes in this layer is symbolized by the symbol S. The input vector’s dimension is equal to the number of nodes, S^55–57^. R is the index of the input layer (*R* = 1, 2,., S), and T is the index of the hidden layer (T = 1, 2,., C). With B = 1, 2,., Z, the output layer’s index is B.

As seen in Fig. 7, the DNN will use these data as a training set. Whereas, the CCA coverage space is divided into 62 sectors; repeat every 2^o^ vertical and horizontal in the interval from − 30^o^ to 30^o^. A 62-bit binary code serves as the input vector for neural networks (one bit for each sector). A (+ 1) bin input denotes the existing main lobe in this sector. The smallest error between the neural model output and the training data is used to determine the connectivity weights. The purpose of the training technique is to fine-tune the weights used for network connections to lower the error function$\:\:\text{E}\text{r}\text{r}\text{o}\text{r}\:\left(\text{g}\right)$, which is defined as:

13 $$\:\text{E}\text{r}\text{r}\text{o}\text{r}\:\left(\text{g}\right)=\frac{1}{2}\sum\:_{\text{B}=1}^{\text{Z}}\sum\:_{\text{T}=1}^{\text{C}}\sum\:_{\text{R}=1}^{\text{S}}{[{\text{y}}_{\text{B}}\left({\text{x}}_{\text{r}},{{\upomega\:}}_{\text{r}\text{t}},{{\upomega\:}}_{\text{b}\text{t}}\right)-{\text{D}\text{a}\text{t}\text{a}}_{\text{B}}]}^{2}\:\:\:$$

Where $\:\text{g}\:=\:1,\:2,\:.\:.\:.\:,\:\text{G}$ is the index of the training set that was obtained from the MGSA-PSO algorithm. This iterative process employs the BP approach discussed in^58^. For each iteration, the weights $\:{{\upomega\:}}_{\text{r}\text{t}}$ and $\:{{\upomega\:}}_{\text{b}\text{t}}$ are modified by:

14 $$\:\varDelta\:{{\upomega\:}}_{\text{v}}=-{\upeta\:}\frac{\partial\:\text{E}\text{r}\text{r}\text{o}\text{r}}{\partial\:{{\upomega\:}}_{\text{v}}}$$

The BP algorithm is a method for training artificial neural networks using gradient descent. They are very powerful and can get extremely complicated. Feedback networks are dynamic; their ‘state’ changes continuously until they reach an equilibrium point. They remain at the equilibrium point until the input changes and a new equilibrium needs to be found^59^. It consists of the following steps:

- Feed the input data through the network and compute the output (the forward pass).
- Compare the output with the desired target and calculate the error.
- Propagate the error backward from the output layer to the input layer, adjusting the weights of each connection according to the gradient of the error for that weight (the backward pass).
- Repeat the process until the error is minimized or a stopping criterion is met.

The BP algorithm is based on the chain rule of calculus, which allows us to compute the derivative of a composite function by multiplying the derivatives of its components. By applying this rule, we can find how much each weight in the network contributes to the error and update it accordingly. In^60^, the BP neural network model was employed to examine the influence of meteorological conditions on hazy weather, considering both univariate and multifactorial aspects. Consequently, the utilization of the BP neural network model for air quality index prediction is considered feasible.

Application developers frequently only have access to a tiny portion of the neural network generation’s potential patterns. Then they make the network, which creates many patterns. For example, the network’s ability to generalize is the most important advantage of neural networks, and this in turn makes the trained network able to classify data that it has not seen before according to the categories of learning data that it has trained on. Also, the calculation scheme for the mean square error behavior of the multi-layer perceptron (MLP) network is shown in Fig. 7.

To achieve this previous generalization in the best way possible. For this, three different sorts of data are utilized. The first of these sets is called the training set and works to reduce the error in the data set to the least possible extent during training. Then comes the second group, which is the validation group, this group works to verify the validity of the data and thus evaluate the success of the neural network on the patterns that were not trained during the training process. The third and final set is a set of tests that work to assess the neural network’s overall effectiveness. In the case of this study, we have two basic steps to get the best performance from the machine learning system as a whole. The first of these two steps is the network configuration step, meaning that first the input vectors are configured {$\:{\text{x}}_{\text{g}}$, $\:\text{g}\:=\:1,\:2,\:.\:.\:.\:,\:60$} which represent the beam-steering angles that correspond to the feeding phase. Input-output pairs are configured, i.e., each input has a corresponding output {$\:{\text{x}}_{\text{g}}$, $\:{{\upphi\:}}_{\text{q}}$}, where $\:\text{q}=\:1,\:2,\:.\:.\:.\:,\:62$, accordingly, it regulates the composition of the whole neural network. As for the second step, which is the network testing step, in this step samples are formed from the vectors that are considered input samples for this stage, and then we present these samples for testing in the neural network designed in the first step. Finally, we get a final output from the network for these input samples. Here, we will notice that the results are strongly, clearly, and non-linearly affected by the number of hidden neurons selected and modeled.

Based on the concepts explained earlier, the type of active neurons tan sigmoid function were used. They are non-linear continuous neurons, and using 45 hidden neurons as shown in Table 2, it was observed that the algorithms converged at a high speed and that the generated neural model was very accurate. One of the main factors that contributed to the accuracy of the final results was the accurate division of the workspace. The workspace has been divided into four main groups: vertical, horizontal, right diagonal, and left diagonal, and each group contains 61 scenarios. Of course, the greater the number of antenna elements, the greater the accuracy of covering the required area by increasing the number of scenarios in each group.

**Table 2:** Typical parameters values used in BP algorithm.

Parameters	Symbol	Value
Neuron in the input layer	$\:\text{a}$	62
Neuron in the output layer	$\:\text{v}$	60
Neuron in the hidden layer	$\:\text{h}$	45
Coefficient of training	$\:{\upeta\:}$	0.03

## Simulated results and discussion

At the beginning of this section, the simulation results for the optimized single antenna element as a building block of the CCA will be presented, analyzed, and compared with the measured results. Then, the design of the CCA is introduced to show its ability to cover difficult terrain roads such as the Taba Ras al-Naqab road in Egypt, with ten beams covering the entire length of the road with all its rugged terrain. Finally, the capability of the applied DNN to cover different bumpy roads is studied.

### Antenna element and CCA design

To resonate the antenna at 2.1 GHz with dual polarization characteristics, the MGSA-PSO algorithm was used to optimize the suggested antenna’s dimensions. The metal circle at the center of the antenna and the ten similar beams that come out were optimized to reach the desired improvement at the assigned band. The suggested antenna is fully investigated using CST-MWS^61^ and then connected with the Matlab-coded optimization technique to improve the antenna dimensions. Accordingly, to reduce the reflection coefficient ($\:{\text{S}}_{11}$), axial ratio ($\:AR$), and maximize antenna gain ($\:\text{G}$) in the appropriate operating frequency band, the below objective function is used.

15 $$\:{\text{O}\text{b}\text{j}}_{2}={\left\{\text{m}\text{i}\text{n}\:\right[{\text{S}}_{11}\:\left(\text{f}\right)\:+\:\text{A}\text{R}\:\left(\text{f}\right)]\:+\text{m}\text{a}\text{x}\:[\text{G}\left(\text{f}\right)\left]\right\}}_{\text{f}=2.1\:\text{G}\text{H}\text{z}}\:\:\:\:\:\:\:\:\:\:\:\:\:\:\:\:\:\:\:\:\:\:\:\:\:$$

**Fig. 8:**
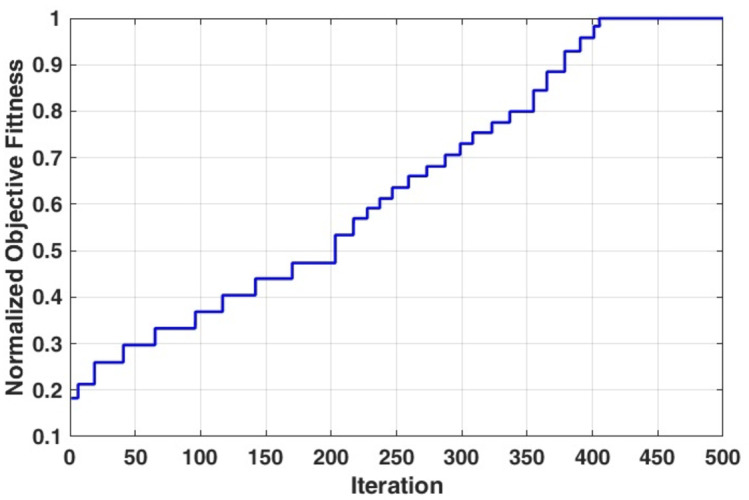
The normalized objective values of the MGSA-PSO algorithm for optimum antenna design.

In such cases, the number of particles and iterations of the MGSA-PSO algorithm are set to be 20 and 500, respectively. Figure 8 shows the normalized objective values convergence capability for optimizing the antenna dimensions. The results demonstrate the ability of the algorithm to adapt its performance throughout the iterations and converge before realizing the maximum number of iterations. The optimum antenna parameters, together with the designated decision space, are listed in Table 3.

**Table 3:** The value of the optimized antenna dimensions (in millimeter).

Variable	Initial value	Decision space		Optimum value (mm)
		From	To	
*R*_*1*_	75	60	80	71.41
*R*_*2*_	55	40	60	49.12
*W*_*1*_	15	12	18	17.05
*W*_*2*_	12	10	15	13.08
*L*_*1*_	20	55	23	18.17
*L*_*2*_	13	5	18	10.15

**Fig. 9:**
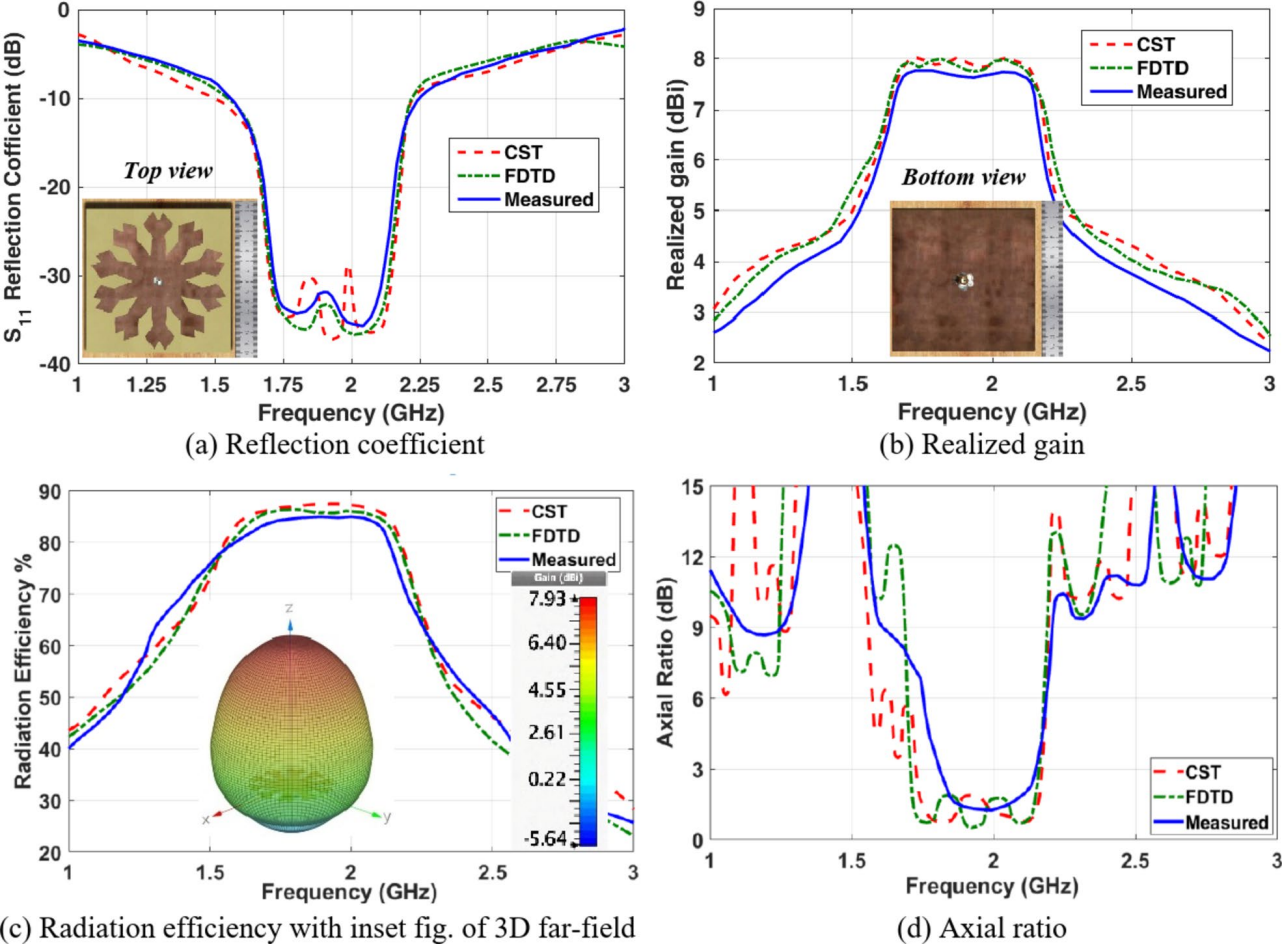
Antenna radiation characteristics at 2.1 GHz.

The antenna is constructed and evaluated using Finite Difference Time Domain (FDTD) Matlab code to confirm the results. In Fig. 9(a) and (b), the measurements of S_11_ and the realized antenna gain, respectively, are shown and compared with the results of the FDTD Matlab code and CST-MWS package, whereas the Measurements are made using an Agilent PNA-L Network Analyzer N5232A, 300 kHz-20 GHz. As depicted in Fig. 9(a), the proposed antenna achieved good matching at the frequency of operation with a measured $\:{\text{S}}_{11}\:$< -33 dB; in addition, the realized gain is measured to be 7.68 dB at 2.1 GHz, as shown in Fig. 9(b). The suggested antenna obtained satisfactory matching at the operating frequency with a measured $\:{\text{S}}_{11}\:$< -33 dB, as shown in Fig. 9(a). Additionally, as shown in Fig. 8(b), the realized gain is measured to be 7.68 dB at 2.1 GHz. In addition, the radiation efficiency is found to be 86.3% at 2.1 GHz, as depicted in Fig. 9(c), with an inset figure of a 3D far-field pattern at 2.1 GHz. The antenna has a broadside radiation pattern and a realized gain of 7.93 dB at 2.1 GHz. Furthermore, as can be seen in Fig. 9(d), the optimized antenna has a considerably improved antenna polarization with an axial ratio of less than 3 dB.

**Fig. 10:**
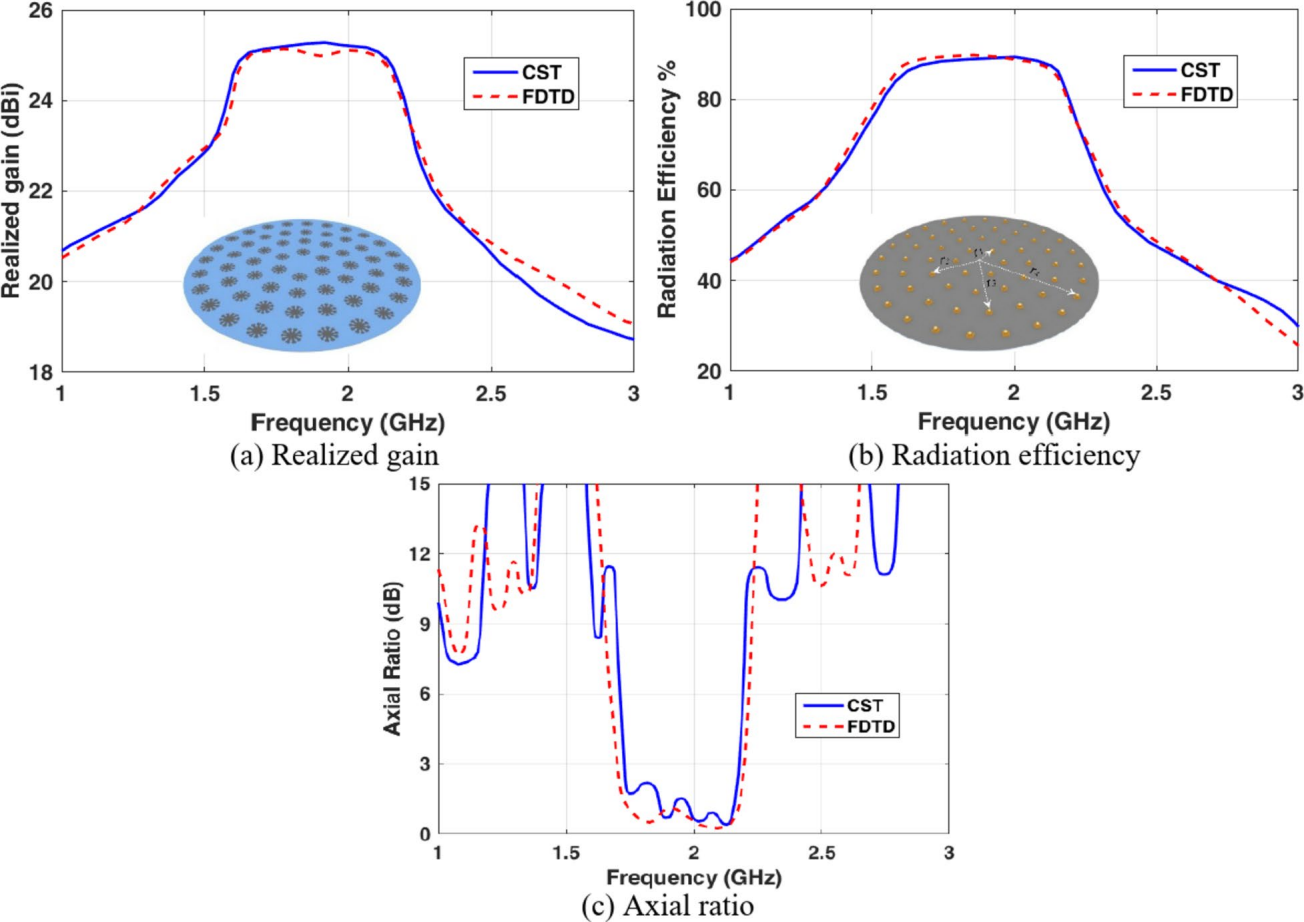
The CCA array radiation characteristics.

The proposed array consists of 60 elements in the configuration of CCA, with four circular rings fed with a total input power of 300 Watts. Figure 10 shows the total realized gain, radiation efficiency, and axial ratio of the CCA. It can be noticed that the CCA realized gain is increased to 25.17 dBi at 2.1 GHz with a radiation efficiency of 87.4% and an axial ratio of 3 dB. The CST-MWS simulation is compared to the FDTD method coded with Matlab to confirm the results, and it can be noticed that there is excellent concordance between the results of the two simulations.

### DNN training scenarios

**Fig. 11:**
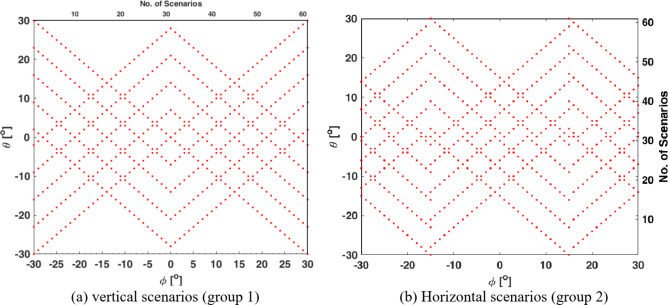
DNN training data of different groups.

There are four main groups to train the DNN system, and these groups are intended to cover the specific ground area of the HAPS, which is equivalent to a square with an area of 645.16 km^2^ and a side length of 25.4 km. Since the HAPS is located at a height of 22 km from the surface of the earth, the required angle to steer the output electromagnetic beams from the HAPS is in the range of -30^o^ to 30^o^ from the center point of the CCA, as shown in Fig. 11. Figure 11 (a) shows the set of scenarios that are considered database training for the neural network in the area to be covered. These scenarios scan the area vertically, so in one scenario there can be one beam, and in another scenario, there can be multiple beams, so we have a large amount of data that contains all possible cases. The same conditions and rules that were applied in the vertical group to generate the database variety were applied to the horizontal group to create the second and horizontal groups, as shown in Fig. 11 (b). The same applies to the third group, in which the beams are titled to the right to treat the winding road tracks on the right, and the fourth group, in which the beams are titled to the left and treat the winding road tracks on the left. The number of scenarios in each group is equivalent to 61 scenarios. Therefore, 244 scenarios are needed to train the HAPS with different angles between horizontal, vertical, right, and left tilt.

**Fig. 12:**
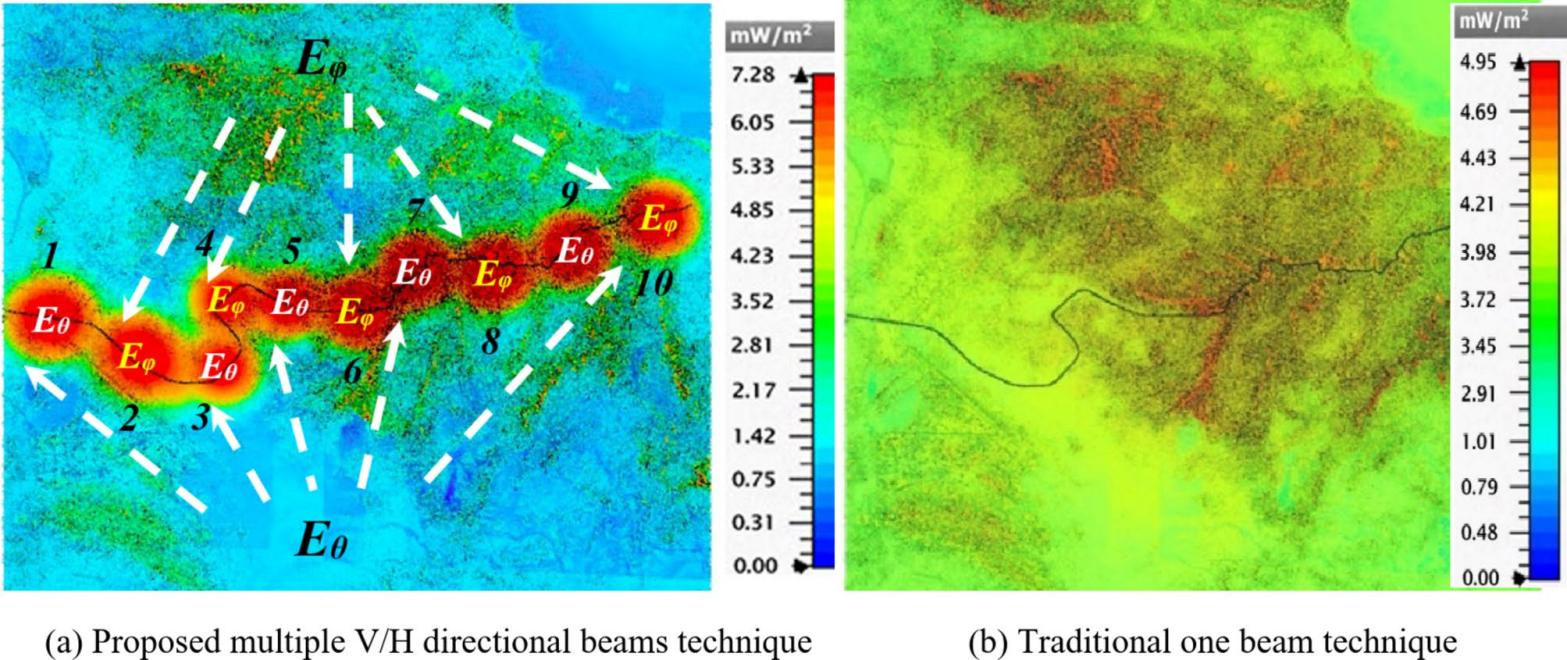
Power density distribution throughout the road path of Taba-Ras al-Naqab training scenario.

As one of the scenarios on which the learning process of this network depends, the Taba-Ras al-Naqab road in the Egypt desert, bounded by difficult terrain, is involved. As shown in Fig. 3(a), the road with an approximated length of 25 km and the HAPS balloon are simulated on the CST-MWS program as described before. Using the MGSA-PSO algorithm, the feeding of the 60-element CCA was controlled. So, the CCA can transmit ten beams at certain angles to cover the tortuous road with high coverage efficiency compared to those obtained using only one beam with a beam width of 60^o^ as shown in Fig. 12(a), whereas each beam has a different polarity than the next one to decrease the interference. It can be noticed the high accuracy of the road coverage from these ten beams compared to those obtained using only one beam with a beam width of 60^o^ as shown in Fig. 12(b). The beam widths of the main beams range from 4^o^ to 8^o^ which can be controlled by placing null angles to the right and left of the main beams. Executing the optimization run with 317 iterations took 1413 min on an x64-based processor (Intel(R) Core (TM) i5-3230 H CPU @ 2.60 GHz, 32 GB RAM) to get the result.

Table 4 introduces the desired and optimized beam directions in addition to the maximum field intensity for each beam. The drift between the desired and obtained directions has not increased by more than 0.7 degrees for elevation and azimuth plans. On the other hand, it should be noted that the power density level throughout the road using the proposed technique ranges from 5.17 to 7.28 mW/m^2^ compared to 4.95 mW/m^2^ as the best value using the traditional technique of single beam.

**Table 4:** Test scenarios for DNN system model.

Beam #	θ^o^		ϕ^o^		Max. field (V/m)
	Desired	Obtained	Desired	Obtained	
1	29	29.87	-2	-2.23	6.79
2	25	25.11	-9	-8.91	6.83
3	20	19.32	-5	-5.64	6.95
4	15	15.21	-1	-0.72	7.08
5	10	9.78	0	0.11	7.15
6	0	0.12	-1	-1.46	7.28
7	-9	-9.36	1	1.68	7.11
8	-17	-16.58	5	4.31	7.04
9	-24	-23.87	7	6.79	6.89
10	-30	-30.15	9	8.83	6.73

### DNN test scenarios for validation

**Fig. 13:**
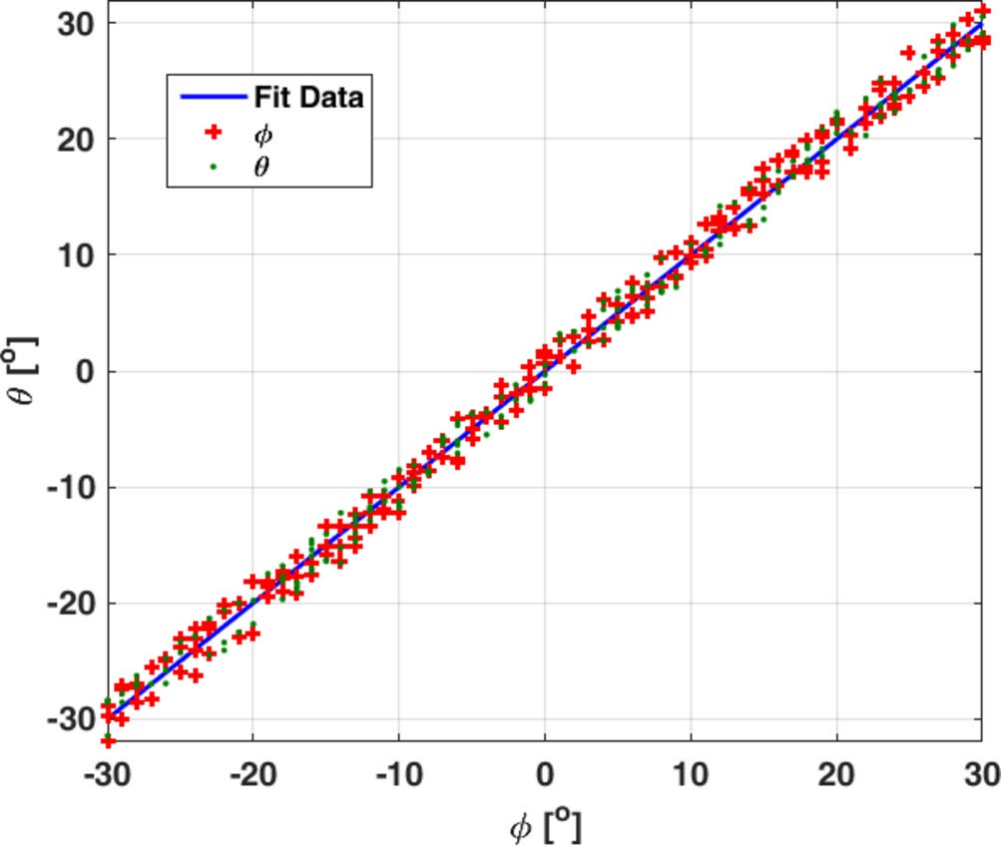
Scatter diagrams of the simulated and computed resonant frequency values by the DNN model for test data.

After training the system with 244 scenarios, including vertical, horizontal, right slash, and left slant, in addition to the Taba-Ras al-Naqab road, the neural network system is tested on 21 scenarios with different angles to ensure the efficiency of the system. The tested scenarios have chosen to steer the pattern at different ten-degree angles from the horizontal and vertical planes to make sure of the ability of the CCA to achieve the test scenario no matter how complex and untrained it is. Figure 13 shows the average error rate that occurred in all test scenarios: APE = 0.213, which is a very small error rate that is almost negligible. The rate of prediction and achievement of the angles to be covered by the artificial intelligence system in the test scenarios is very high. In each scenario, the amplitude is assumed to be uniform for all antenna elements, while the neural network is responsible for producing 60 feeding phases that produce multi-beams to cover the HAPS road path.

**Fig. 14:**
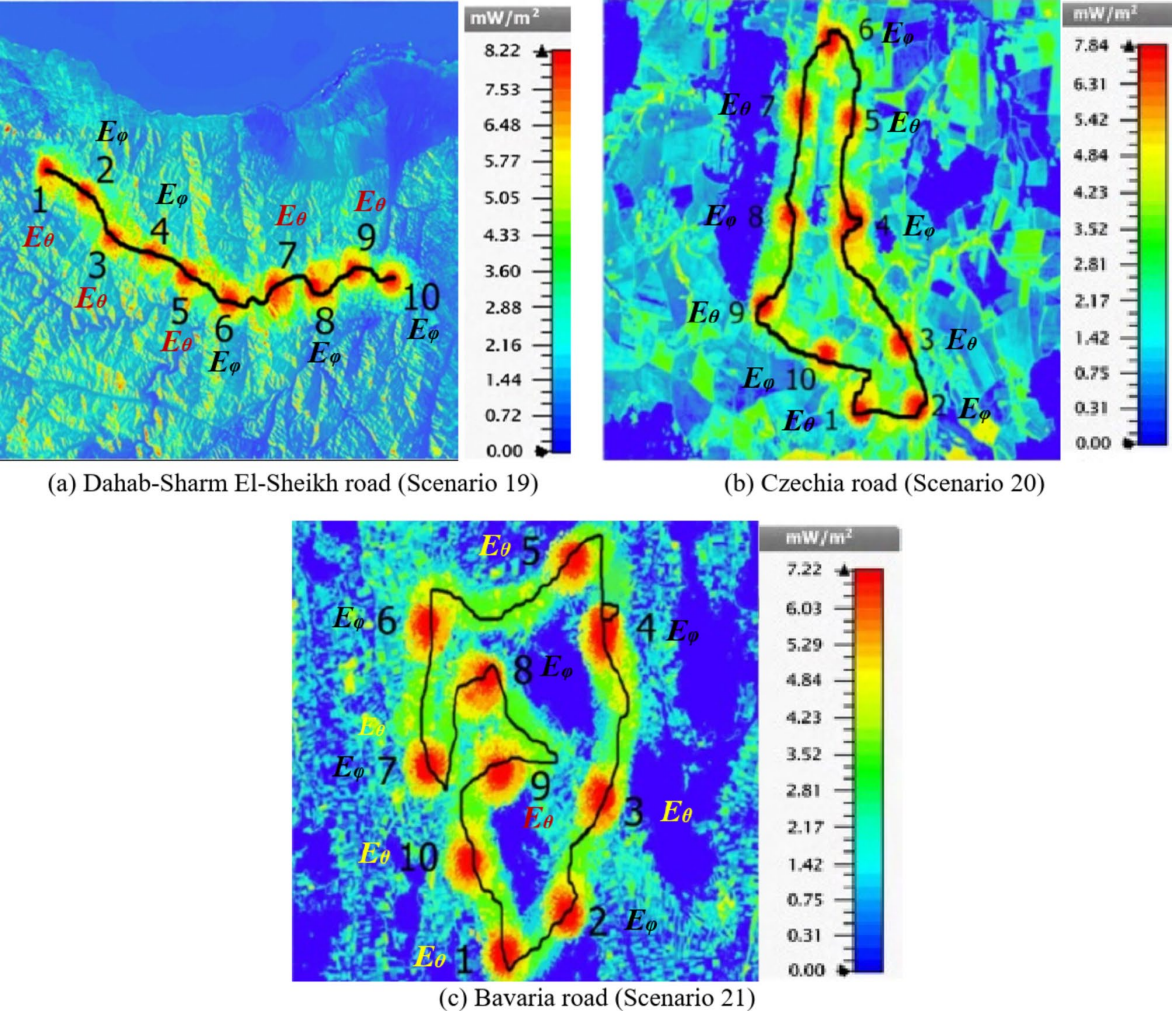
Power density distribution throughout the road path for different test scenarios.

Among these test scenarios, three are presented and discussed as real geographic areas with different terrain characteristics. Firstly, a bumpy road with mountain chains, such as the Dahab-Sharm El-Sheikh road in Egypt, is introduced. Then, two other roads, such as those in Czechia and Bavaria in Germany, are considered areas where forests and trees abound. As shown in Fig. 14, the DNN has assigned 10 beams to cover these different roads, whose lengths are approximately 25 km, 22.81 km, and 57.38 km, respectively. The simulated power density throughout the road ranges between 5 and 8.22 mW/m^2^ which is an acceptable value for mobile devices^62^. The results show the capability of DNN to cover the Dahab-Sharm El-Sheikh, Czechia, and Bavaria roads with 10 beam patterns, as shown in Fig. 14(a, b, and c), respectively. The obtained results show the power density distribution along the Dahab-Sharm El-Sheikh, Czechia, and Bavaria roads, with intensity values ranging from 3.6 to 8.22 mW/m^2^, from 3.5 to 7.84 mW/m^2^ and from 2.8 to 7.22 mW/m^2^, respectively.

Table 5 introduces the desired directions and the obtained beam directions from DNN, in addition to the maximum field intensity for each beam. It is clear that the drift between the desired and obtained directions has not increased more than 3.6^o^ and 2.5^o^ for elevation and azimuth plans, respectively. Furthermore, the maximum field intensity of different steered beams for the tested scenarios ranged from 6.4 to 7.5 V/m. Actually, the proposed technique can be used as a generic solution to cover any track path just by determining the road coordinates. Adaptively correcting the footprint on the ground by changing the direction of radio beams as the HAP spins using DNN by changing the road path for any reason, the beam pattern direction will be adapted to cover the new path. Concerning the radiated power from the HAPS antenna array into the desired directions, increasing the received power and hence increasing the bit rates and throughput. Furthermore, it can save the required power.

**Fig. 15:**
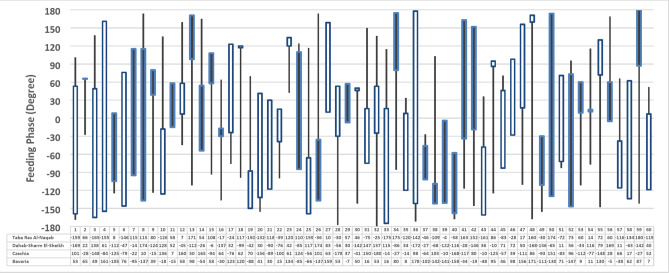
Feeding phases for the 60 elements for each different proposed scenarios.

To illustrate the dynamics of the feeding phase for the different spots, Fig. 15 is presented to show the feeding phases in the 60 elements for each scenario. The feeding phase can change from − 180^o^ to 180^o^. Recently, innovations in integrated circuit design and processes have made the benefits of phased array technology available. For example, IBM and Ericsson announced the world’s first reported Si mmWave phased-array antenna module, consisting of four MMICs and 64 dual-polarized antennas for concurrent dual-polarization operation and 1.4° beam-steering resolution for 5G communication^63^.

**Table 5:** A comparison between the desired and the obtained beam directions from DNN for different test scenarios, in addition to the maximum field intensity for each beam and the time consumption.

Test scenario	Beam no.	θ^o^		ϕ^o^		Max. field (V/m)	Time consuming (min)
		Des.	Obt.	Des.	Obt.		
Dahab-Sharm Elsheikh	1	30	29.2	8	9.1	6.4	2.3
	2	23	23.7	4	4.5	6.6	
	3	18	18.6	-2	-2.9	6.5	
	4	12	12.8	-4	-4.4	7.2	
	5	6	6.9	-7.5	-7.7	7.0	
	6	-2	-3.1	-12	-12.5	6.7	
	7	-9	-9.8	-10	-10.3	7.3	
	8	-17	-17.7	-10	-9.6	7.4	
	9	-22	-23.1	-6	-7.5	6.4	
	10	-30	-28.9	-7	-8.1	7.1	
Czechia	1	-30	-33.6	-4	-3.7	6.8	2.5
	2	-26	-27.5	7	8.1	7.1	
	3	-7	-7.6	12.5	13.6	6.7	
	4	20	20.7	14	13.4	7.3	
	5	30	32.5	8	7.5	6.5	
	6	22	22.6	-17.5	-17.7	6.9	
	7	-2.5	-2.1	-17.5	-16.8	7.4	
	8	12.5	13.2	-2	-2.9	7.5	
	9	-3.5	-3.1	-3	-3.8	6.6	
	10	-17.5	-18.9	-10	-9.1	7.2	
Bavaria	1	-30	-32.1	5	4.1	7.4	2.2
	2	-30	-31.6	14	13.4	6.7	
	3	-20	-20.3	12.5	12.1	7.0	
	4	2	2.4	2.5	3.0	7.5	
	5	17.5	18.4	3	3.3	6.9	
	6	30	32.2	0	2.1	6.8	
	7	20	-21.9	-5	-6.2	7.1	
	8	2.5	1.6	-7.5	-7.4	7.2	
	9	-12.5	-13.1	-12.5	-12.0	6.6	
	10	-22.5	-21.7	-2.5	-1.6	7.3	

Other than the potential applications and impact of traditional HAPs, the proposed approach is a generic solution to cover different areas with different topologies by adapting the beam direction based on DNN. As a result, it can be used to support a variety of use cases for both developed and developing markets, especially for covering certain paths in rural and difficult, rugged zones. On the other hand, the proposed technique has some limitations associated with the current work that direct the future research direction. These limitations are listed as indicated below:

#### Multiband operation

The current design operates at a single frequency (2.1 GHz), limiting its versatility. Future work should enable the synthesis of multiple V/H directional beams across different frequency bands. This will enhance the system’s robustness and applicability to emerging technologies such as the Internet of Things (IoT) and 6G networks, which often require multiband support.

#### Alternative antenna geometries

The current work employs a concentric circular array (CCA) geometry. Future research should investigate alternative geometries, such as conical, and cylindrical arrays, or hybrid array configurations, such as non-uniform arrays, to enhance design flexibility and improve beam synthesis performance, particularly in complex terrains. These alternative geometries may offer advantages in terms of gain, coverage, and adaptability.

#### Scalability to larger arrays with reduced complexity

Future research should explore strategies for reducing the number of elements while maintaining acceptable performance. This could involve investigating more efficient optimization techniques, lightweight algorithms.

#### Adaptability to dynamic environments

The current beamforming system is designed for static road scenarios. In addition, the impact of atmospheric conditions and interference from other sources on HAPS performance is not explicitly addressed. Future work should incorporate real-time adaptability, enabling the system to dynamically adjust beam patterns and feeding phases in response to changes in environmental factors (e.g., extreme weather, high interference zones). This could be achieved through advanced deep reinforcement learning or adaptive algorithms.

#### Extended validation in real-world scenarios

The proposed technique has been primarily validated through simulations. Further experimental validation in real-world scenarios is crucial to address practical challenges such as interference, hardware constraints, and deployment challenges. Adding tests that evaluate robustness under diverse conditions or unexpected challenges would bolster confidence in the model’s adaptability and reliability.)

#### Integration with mobility management in HAPS

The current study does not account for the dynamic movement of HAPS. Future research should investigate the integration of mobility management techniques, allowing for real-time adjustments to altitude, trajectory, and beam steering to optimize signal coverage and ensure seamless connectivity.

#### Power density and health considerations

While the study ensures acceptable power density levels for mobile devices, future work should further investigate compliance with international safety standards (e.g., ICNIRP guidelines) and explore power optimization techniques to minimize energy consumption while maintaining system performance.

By addressing these directions, the proposed system can overcome the limitations of the current work and move closer to practical, scalable, and robust deployment for next-generation HAPS communications.

## Conclusion

The integration of High-Altitude Platforms (HAPs) with Deep Learning (DL) models presents a promising avenue for revolutionizing telecommunications by enabling low-cost and scalable service delivery. DL models offer the potential to significantly enhance HAP performance by optimizing beamforming through intelligent altitude and trajectory adjustments, facilitating the analysis of vast datasets, and improving overall system performance. This synergistic approach will be instrumental in supporting emerging technologies such as the Internet of Things (IoT) and 6G, while also providing more reliable and robust communication services to address the ever-growing demand for data and connectivity. This paper introduces a novel Concentric Circular Array (CCA) for HAPS communications, designed to generate multiple high-gain beams to effectively cover designated road paths. The study begins with a thorough characterization of the individual antenna elements, comparing simulated and measured results to validate the design. Subsequently, the paper presents the simulated performance of the CCA, demonstrating its capability to effectively cover extended road lengths using ten distinct beams. Notably, the proposed CCA design leverages polarization and directivity control to synthesize multiple vertical/horizontal directional beams.

Furthermore, the paper explores the application of a Deep Neural Network (DNN) to optimize beamforming patterns for different road paths by determining the optimal feeding phases for each CCA element. The achieved power density levels across various road paths, ranging from 5 to 8.21 mW/m², are deemed suitable for mobile device operation. Future research directions include investigating the applicability of the proposed model to alternative frequency bands, such as 5G and mmWave frequencies, and exploring methods to mitigate interference, particularly in challenging urban fringe environments. Additionally, exploring alternative antenna array geometries, such as non-uniform circular arrays, conical configurations, or cylindrical configurations, can further enhance the system’s performance and coverage capabilities.

## Methods

To analyze the performance of the entire structure, a 3D full-wave numerical simulation was performed using Matlab to control commercial electromagnetic software such as CST-MWS. The interface between Matlab and CST-MWS is created via a scripting language called Visual Basic for Applications (VBA script). To Determine the area that needs to be covered, the Google Earth program is used to assign its longitude and latitude coordinates. Then, the coordinates of the boundaries of the region from all directions are assigned by the Earth Explorer (EE) user interface tool developed by the United States Geological Survey (USGS). Finally, the DNN-based approach in Matlab is applied for HAPS multi-beam beamforming design.

## Data Availability

The data sets used and/or analyzed during the current investigation are provided within the manuscript. More data sets will be available upon reasonable request from the corresponding author.
